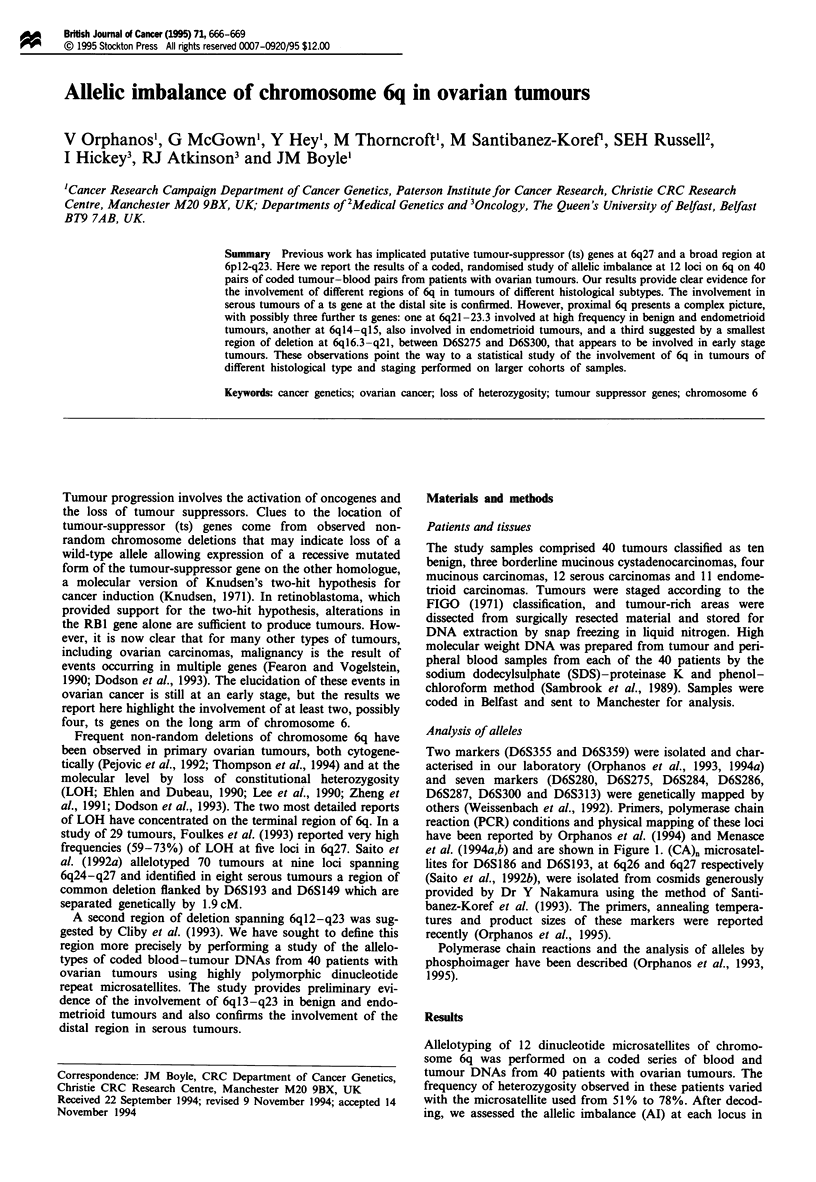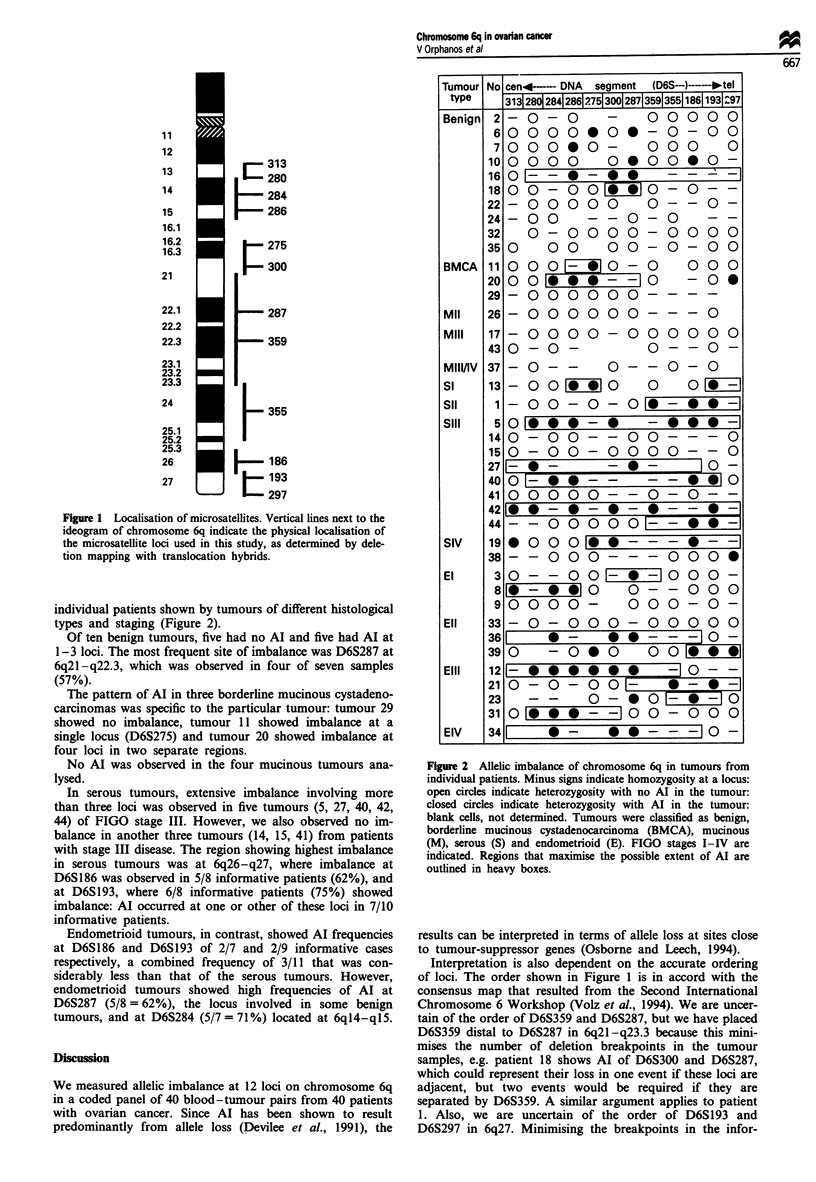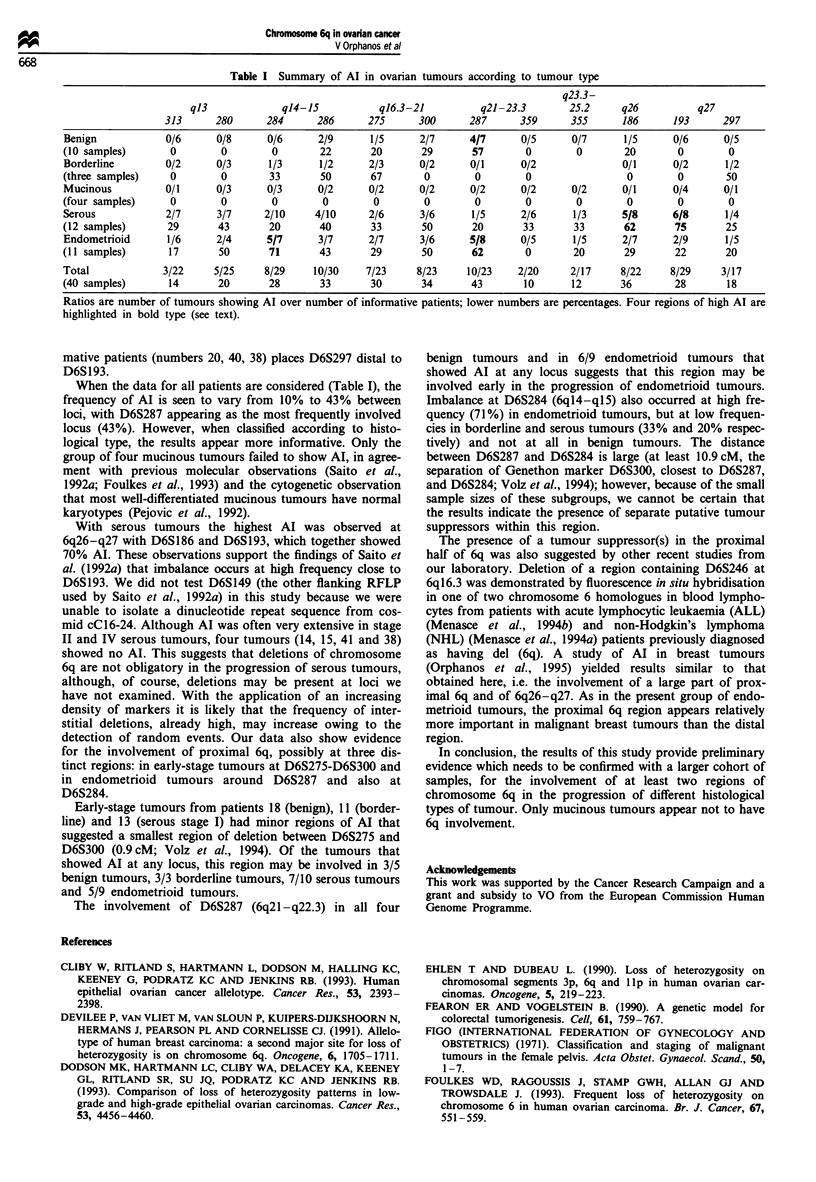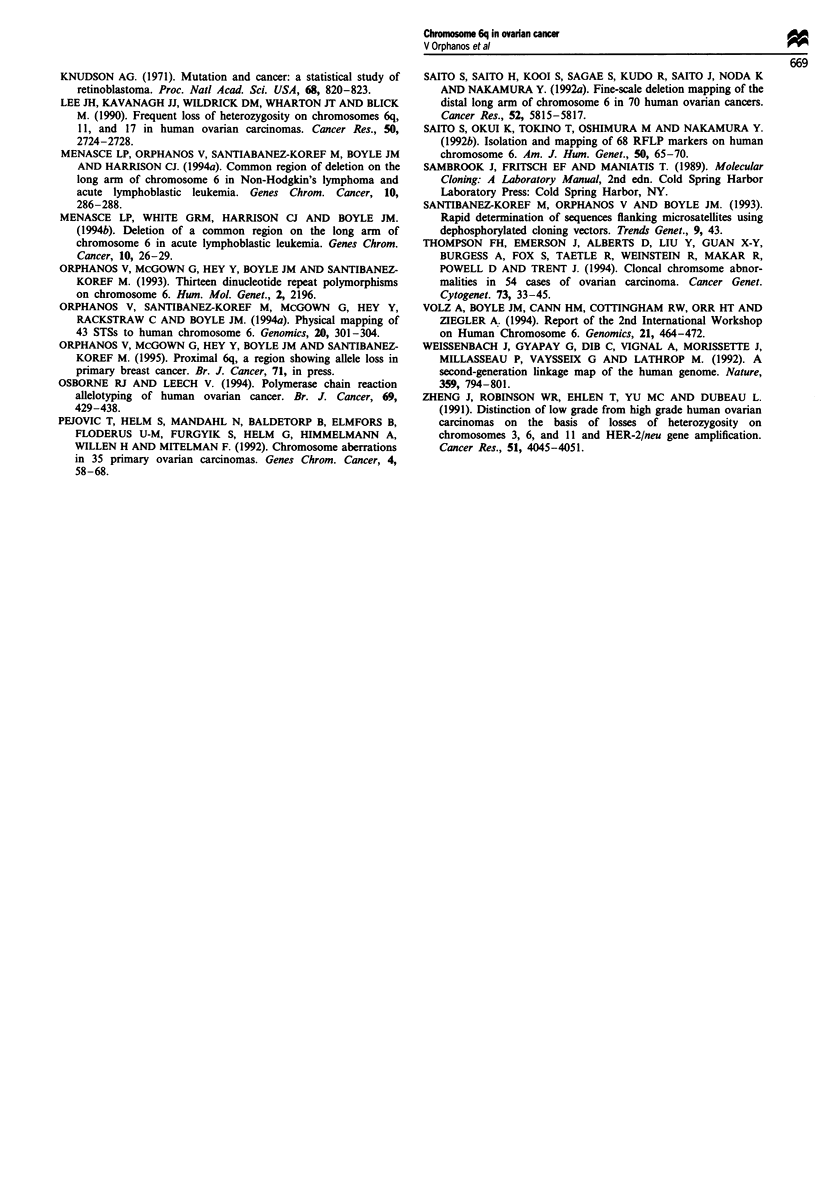# Allelic imbalance of chromosome 6q in ovarian tumours.

**DOI:** 10.1038/bjc.1995.132

**Published:** 1995-04

**Authors:** V. Orphanos, G. McGown, Y. Hey, M. Thorncroft, M. Santibanez-Koref, S. E. Russell, I. Hickey, R. J. Atkinson, J. M. Boyle

**Affiliations:** Department of Cancer Genetics, Paterson Institute for Cancer Research, Christie CRC Research Centre, Manchester, UK.

## Abstract

Previous work has implicated putative tumour-suppressor (ts) genes at 6q27 and a broad region at 6p12-q23. Here we report the results of a coded, randomised study of allelic imbalance at 12 loci on 6q on 40 pairs of coded tumour-blood pairs from patients with ovarian tumours. Our results provide clear evidence for the involvement of different regions of 6q in tumours of different histological subtypes. The involvement in serous tumours of a ts gene at the distal site is confirmed. However, proximal 6q presents a complex picture, with possibly three further ts genes: one at 6q21-23.3 involved at high frequency in benign and endometrioid tumours, another at 6q14-q15, also involved in endometrioid tumours, and a third suggested by a smallest region of deletion at 6q16.3-q21, between D6S275 and D6S300, that appears to be involved in early stage tumours. These observations point the way to a statistical study of the involvement of 6q in tumours of different histological type and staging performed on larger cohorts of samples.


British Journal of Cancer (1995) 71, 666-669
© 1995 Stockton Press All rights reserved 0007-0920/95 $12.00

### Allelic imbalance of chromosome 6q in ovarian tumours

V Orphanos[1], G McGown[1], Y Hey[1], M Thorncroft[1], M Santibanez-Koref[1], SEH Russell[2], I Hickey[3], RJ Atkinson[3] and JM Boyle[1]

[1]*Cancer Research Campaign Department of Cancer Genetics, Paterson Institute for Cancer Research, Christie CRC Research Centre, Manchester M20 9BX, UK; Departments of* [2]*Medical Genetics and* [3]*Oncology, The Queen's University of Belfast, Belfast BT9 7AB, UK.*

**Summary** Previous work has implicated putative tumour-suppressor (ts) genes at 6q27 and a broad region at 6p12-q23. Here we report the results of a coded, randomised study of allelic imbalance at 12 loci on 6q on 40 pairs of coded tumour-blood pairs from patients with ovarian tumours. Our results provide clear evidence for the involvement of different regions of 6q in tumours of different histological subtypes. The involvement in serous tumours of a ts gene at the distal site is confirmed. However, proximal 6q presents a complex picture, with possibly three further ts genes: one at 6q21-23.3 involved at high frequency in benign and endometrioid tumours, another at 6q14-q15, also involved in endometrioid tumours, and a third suggested by a smallest region of deletion at 6q16.3-q21, between D6S275 and D6S300, that appears to be involved in early stage tumours. These observations point the way to a statistical study of the involvement of 6q in tumours of different histological type and staging performed on larger cohorts of samples.

**Keywords:** cancer genetics; ovarian cancer; loss of heterozygosity; tumour suppressor genes; chromosome 6

Tumour progression involves the activation of oncogenes and the loss of tumour suppressors. Clues to the location of tumour-suppressor (ts) genes come from observed non-random chromosome deletions that may indicate loss of a wild-type allele allowing expression of a recessive mutated form of the tumour-suppressor gene on the other homologue, a molecular version of Knudsen's two-hit hypothesis for cancer induction (Knudsen, 1971). In retinoblastoma, which provided support for the two-hit hypothesis, alterations in the RB1 gene alone are sufficient to produce tumours. However, it is now clear that for many other types of tumours, including ovarian carcinomas, malignancy is the result of events occurring in multiple genes (Fearon and Vogelstein, 1990; Dodson *et al.*, 1993). The elucidation of these events in ovarian cancer is still at an early stage, but the results we report here highlight the involvement of at least two, possibly four, ts genes on the long arm of chromosome 6.

Frequent non-random deletions of chromosome 6q have been observed in primary ovarian tumours, both cytogenetically (Pejovic *et al.*, 1992; Thompson *et al.*, 1994) and at the molecular level by loss of constitutional heterozygosity (LOH; Ehlen and Dubeau, 1990; Lee *et al.*, 1990; Zheng *et al.*, 1991; Dodson *et al.*, 1993). The two most detailed reports of LOH have concentrated on the terminal region of 6q. In a study of 29 tumours, Foulkes *et al.* (1993) reported very high frequencies (59-73%) of LOH at five loci in 6q27. Saito *et al.* (1992*a*) allelotyped 70 tumours at nine loci spanning 6q24-q27 and identified in eight serous tumours a region of common deletion flanked by D6S193 and D6S149 which are separated genetically by 1.9 cM.

A second region of deletion spanning 6q12-q23 was suggested by Cliby *et al.* (1993). We have sought to define this region more precisely by performing a study of the allelotypes of coded blood-tumour DNAs from 40 patients with ovarian tumours using highly polymorphic dinucleotide repeat microsatellites. The study provides preliminary evidence of the involvement of 6q13-q23 in benign and endometrioid tumours and also confirms the involvement of the distal region in serous tumours.

Correspondence: JM Boyle, CRC Department of Cancer Genetics, Christie CRC Research Centre, Manchester M20 9BX, UK
Received 22 September 1994; revised 9 November 1994; accepted 14 November 1994

### Materials and methods

### *Patients and tissues*

The study samples comprised 40 tumours classified as ten benign, three borderline mucinous cystadenocarcinomas, four mucinous carcinomas, 12 serous carcinomas and 11 endometrioid carcinomas. Tumours were staged according to the FIGO (1971) classification, and tumour-rich areas were dissected from surgically resected material and stored for DNA extraction by snap freezing in liquid nitrogen. High molecular weight DNA was prepared from tumour and peripheral blood samples from each of the 40 patients by the sodium dodecylsulphate (SDS)-proteinase K and phenol-chloroform method (Sambrook *et al.*, 1989). Samples were coded in Belfast and sent to Manchester for analysis.

### *Analysis of alleles*

Two markers (D6S355 and D6S359) were isolated and characterised in our laboratory (Orphanos *et al.*, 1993, 1994*a*) and seven markers (D6S280, D6S275, D6S284, D6S286, D6S287, D6S300 and D6S313) were genetically mapped by others (Weissenbach *et al.*, 1992). Primers, polymerase chain reaction (PCR) conditions and physical mapping of these loci have been reported by Orphanos *et al.* (1994) and Menasce *et al.* (1994*a,b*) and are shown in Figure 1. $(CA)_n$ microsatellites for D6S186 and D6S193, at 6q26 and 6q27 respectively (Saito *et al.*, 1992*b*), were isolated from cosmids generously provided by Dr Y Nakamura using the method of Santibanez-Koref *et al.* (1993). The primers, annealing temperatures and product sizes of these markers were reported recently (Orphanos *et al.*, 1995).

Polymerase chain reactions and the analysis of alleles by phosphoimager have been described (Orphanos *et al.*, 1993, 1995).

### Results

Allelotyping of 12 dinucleotide microsatellites of chromosome 6q was performed on a coded series of blood and tumour DNAs from 40 patients with ovarian tumours. The frequency of heterozygosity observed in these patients varied with the microsatellite used from 51% to 78%. After decoding, we assessed the allelic imbalance (AI) at each locus in

**Figure 1** Localisation of microsatellites. Vertical lines next to the ideogram of chromosome 6q indicate the physical localisation of the microsatellite loci used in this study, as determined by deletion mapping with translocation hybrids.

individual patients shown by tumours of different histological types and staging (Figure 2).

Of ten benign tumours, five had no AI and five had AI at 1–3 loci. The most frequent site of imbalance was D6S287 at 6q21–q22.3, which was observed in four of seven samples (57%).

The pattern of AI in three borderline mucinous cystadenocarcinomas was specific to the particular tumour: tumour 29 showed no imbalance, tumour 11 showed imbalance at a single locus (D6S275) and tumour 20 showed imbalance at four loci in two separate regions.

No AI was observed in the four mucinous tumours analysed.

In serous tumours, extensive imbalance involving more than three loci was observed in five tumours (5, 27, 40, 42, 44) of FIGO stage III. However, we also observed no imbalance in another three tumours (14, 15, 41) from patients with stage III disease. The region showing highest imbalance in serous tumours was at 6q26–q27, where imbalance at D6S186 was observed in 5/8 informative patients (62%), and at D6S193, where 6/8 informative patients (75%) showed imbalance: AI occurred at one or other of these loci in 7/10 informative patients.

Endometrioid tumours, in contrast, showed AI frequencies at D6S186 and D6S193 of 2/7 and 2/9 informative cases respectively, a combined frequency of 3/11 that was considerably less than that of the serous tumours. However, endometrioid tumours showed high frequencies of AI at D6S287 (5/8 = 62%), the locus involved in some benign tumours, and at D6S284 (5/7 = 71%) located at 6q14–q15.

### Discussion

We measured allelic imbalance at 12 loci on chromosome 6q in a coded panel of 40 blood–tumour pairs from 40 patients with ovarian cancer. Since AI has been shown to result predominantly from allele loss (Devilee et al., 1991), the

**Figure 2** Allelic imbalance of chromosome 6q in tumours from individual patients. Minus signs indicate homozygosity at a locus: open circles indicate heterozygosity with no AI in the tumour: closed circles indicate heterozygosity with AI in the tumour: blank cells, not determined. Tumours were classified as benign, borderline mucinous cystadenocarcinoma (BMCA), mucinous (M), serous (S) and endometrioid (E). FIGO stages I–IV are indicated. Regions that maximise the possible extent of AI are outlined in heavy boxes.

results can be interpreted in terms of allele loss at sites close to tumour-suppressor genes (Osborne and Leech, 1994).

Interpretation is also dependent on the accurate ordering of loci. The order shown in Figure 1 is in accord with the consensus map that resulted from the Second International Chromosome 6 Workshop (Volz et al., 1994). We are uncertain of the order of D6S359 and D6S287, but we have placed D6S359 distal to D6S287 in 6q21–q23.3 because this minimises the number of deletion breakpoints in the tumour samples, e.g. patient 18 shows AI of D6S300 and D6S287, which could represent their loss in one event if these loci are adjacent, but two events would be required if they are separated by D6S359. A similar argument applies to patient 1. Also, we are uncertain of the order of D6S193 and D6S297 in 6q27. Minimising the breakpoints in the infor-

**Table I** Summary of AI in ovarian tumours according to tumour type

| | *q13* | | *q14–15* | | *q16.3–21* | | *q21–23.3* | | *q23.3–25.2* | *q26* | *q27* | |
|---|---|---|---|---|---|---|---|---|---|---|---|---|
| | *313* | *280* | *284* | *286* | *275* | *300* | *287* | *359* | *355* | *186* | *193* | *297* |
| Benign | 0/6 | 0/8 | 0/6 | 2/9 | 1/5 | 2/7 | **4/7** | 0/5 | 0/7 | 1/5 | 0/6 | 0/5 |
| (10 samples) | 0 | 0 | 0 | 22 | 20 | 29 | **57** | 0 | 0 | 20 | 0 | 0 |
| Borderline | 0/2 | 0/3 | 1/3 | 1/2 | 2/3 | 0/2 | 0/1 | 0/2 | | 0/1 | 0/2 | 1/2 |
| (three samples) | 0 | 0 | 33 | 50 | 67 | 0 | 0 | 0 | | 0 | 0 | 50 |
| Mucinous | 0/1 | 0/3 | 0/3 | 0/2 | 0/2 | 0/2 | 0/2 | 0/2 | 0/2 | 0/1 | 0/4 | 0/1 |
| (four samples) | 0 | 0 | 0 | 0 | 0 | 0 | 0 | 0 | 0 | 0 | 0 | 0 |
| Serous | 2/7 | 3/7 | 2/10 | 4/10 | 2/6 | 3/6 | 1/5 | 2/6 | 1/3 | **5/8** | **6/8** | 1/4 |
| (12 samples) | 29 | 43 | 20 | 40 | 33 | 50 | 20 | 33 | 33 | **62** | **75** | 25 |
| Endometrioid | 1/6 | 2/4 | **5/7** | 3/7 | 2/7 | 3/6 | **5/8** | 0/5 | 1/5 | 2/7 | 2/9 | 1/5 |
| (11 samples) | 17 | 50 | **71** | 43 | 29 | 50 | **62** | 0 | 20 | 29 | 22 | 20 |
| Total | 3/22 | 5/25 | 8/29 | 10/30 | 7/23 | 8/23 | 10/23 | 2/20 | 2/17 | 8/22 | 8/29 | 3/17 |
| (40 samples) | 14 | 20 | 28 | 33 | 30 | 34 | 43 | 10 | 12 | 36 | 28 | 18 |

Ratios are number of tumours showing AI over number of informative patients; lower numbers are percentages. Four regions of high AI are highlighted in bold type (see text).

mative patients (numbers 20, 40, 38) places D6S297 distal to D6S193.

When the data for all patients are considered (Table I), the frequency of AI is seen to vary from 10% to 43% between loci, with D6S287 appearing as the most frequently involved locus (43%). However, when classified according to histological type, the results appear more informative. Only the group of four mucinous tumours failed to show AI, in agreement with previous molecular observations (Saito *et al.*, 1992*a*; Foulkes *et al.*, 1993) and the cytogenetic observation that most well-differentiated mucinous tumours have normal karyotypes (Pejovic *et al.*, 1992).

With serous tumours the highest AI was observed at 6q26–q27 with D6S186 and D6S193, which together showed 70% AI. These observations support the findings of Saito *et al.* (1992*a*) that imbalance occurs at high frequency close to D6S193. We did not test D6S149 (the other flanking RFLP used by Saito *et al.*, 1992*a*) in this study because we were unable to isolate a dinucleotide repeat sequence from cosmid cC16-24. Although AI was often very extensive in stage II and IV serous tumours, four tumours (14, 15, 41 and 38) showed no AI. This suggests that deletions of chromosome 6q are not obligatory in the progression of serous tumours, although, of course, deletions may be present at loci we have not examined. With the application of an increasing density of markers it is likely that the frequency of interstitial deletions, already high, may increase owing to the detection of random events. Our data also show evidence for the involvement of proximal 6q, possibly at three distinct regions: in early-stage tumours at D6S275-D6S300 and in endometrioid tumours around D6S287 and also at D6S284.

Early-stage tumours from patients 18 (benign), 11 (borderline) and 13 (serous stage I) had minor regions of AI that suggested a smallest region of deletion between D6S275 and D6S300 (0.9 cM; Volz *et al.*, 1994). Of the tumours that showed AI at any locus, this region may be involved in 3/5 benign tumours, 3/3 borderline tumours, 7/10 serous tumours and 5/9 endometrioid tumours.

The involvement of D6S287 (6q21–q22.3) in all four benign tumours and in 6/9 endometrioid tumours that showed AI at any locus suggests that this region may be involved early in the progression of endometrioid tumours. Imbalance at D6S284 (6q14–q15) also occurred at high frequency (71%) in endometrioid tumours, but at low frequencies in borderline and serous tumours (33% and 20% respectively) and not at all in benign tumours. The distance between D6S287 and D6S284 is large (at least 10.9 cM, the separation of Genethon marker D6S300, closest to D6S287, and D6S284; Volz *et al.*, 1994); however, because of the small sample sizes of these subgroups, we cannot be certain that the results indicate the presence of separate putative tumour suppressors within this region.

The presence of a tumour suppressor(s) in the proximal half of 6q was also suggested by other recent studies from our laboratory. Deletion of a region containing D6S246 at 6q16.3 was demonstrated by fluorescence *in situ* hybridisation in one of two chromosome 6 homologues in blood lymphocytes from patients with acute lymphocytic leukaemia (ALL) (Menasce *et al.*, 1994*b*) and non-Hodgkin's lymphoma (NHL) (Menasce *et al.*, 1994*a*) patients previously diagnosed as having del (6q). A study of AI in breast tumours (Orphanos *et al.*, 1995) yielded results similar to that obtained here, i.e. the involvement of a large part of proximal 6q and of 6q26–q27. As in the present group of endometrioid tumours, the proximal 6q region appears relatively more important in malignant breast tumours than the distal region.

In conclusion, the results of this study provide preliminary evidence which needs to be confirmed with a larger cohort of samples, for the involvement of at least two regions of chromosome 6q in the progression of different histological types of tumour. Only mucinous tumours appear not to have 6q involvement.

**Acknowledgements**
This work was supported by the Cancer Research Campaign and a grant and subsidy to VO from the European Commission Human Genome Programme.

### References

CLIBY W, RITLAND S, HARTMANN L, DODSON M, HALLING KC, KEENEY G, PODRATZ KC AND JENKINS RB. (1993). Human epithelial ovarian cancer allelotype. *Cancer Res.*, **53**, 2393–2398.

DEVILEE P, VAN VLIET M, VAN SLOUN P, KUIPERS-DIJKSHOORN N, HERMANS J, PEARSON PL AND CORNELISSE CJ. (1991). Allelotype of human breast carcinoma: a second major site for loss of heterozygosity is on chromosome 6q. *Oncogene*, **6**, 1705–1711.

DODSON MK, HARTMANN LC, CLIBY WA, DELACEY KA, KEENEY GL, RITLAND SR, SU JQ, PODRATZ KC AND JENKINS RB. (1993). Comparison of loss of heterozygosity patterns in low-grade and high-grade epithelial ovarian carcinomas. *Cancer Res.*, **53**, 4456–4460.

EHLEN T AND DUBEAU L. (1990). Loss of heterozygosity on chromosomal segments 3p, 6q and 11p in human ovarian carcinomas. *Oncogene*, **5**, 219–223.

FEARON ER AND VOGELSTEIN B. (1990). A genetic model for colorectal tumorigenesis. *Cell*, **61**, 759–767.

FIGO (INTERNATIONAL FEDERATION OF GYNECOLOGY AND OBSTETRICS) (1971). Classification and staging of malignant tumours in the female pelvis. *Acta Obstet. Gynaecol. Scand.*, **50**, 1–7.

FOULKES WD, RAGOUSSIS J, STAMP GWH, ALLAN GJ AND TROWSDALE J. (1993). Frequent loss of heterozygosity on chromosome 6 in human ovarian carcinoma. *Br. J. Cancer*, **67**, 551–559.

KNUDSON AG. (1971). Mutation and cancer: a statistical study of retinoblastoma. *Proc. Natl Acad. Sci. USA*, **68**, 820-823.

LEE JH, KAVANAGH JJ, WILDRICK DM, WHARTON JT AND BLICK M. (1990). Frequent loss of heterozygosity on chromosomes 6q, 11, and 17 in human ovarian carcinomas. *Cancer Res.*, **50**, 2724-2728.

MENASCE LP, ORPHANOS V, SANTIABANEZ-KOREF M, BOYLE JM AND HARRISON CJ. (1994*a*). Common region of deletion on the long arm of chromosome 6 in Non-Hodgkin's lymphoma and acute lymphoblastic leukemia. *Genes Chrom. Cancer*, **10**, 286-288.

MENASCE LP, WHITE GRM, HARRISON CJ AND BOYLE JM. (1994*b*). Deletion of a common region on the long arm of chromosome 6 in acute lymphoblastic leukemia. *Genes Chrom. Cancer*, **10**, 26-29.

ORPHANOS V, MCGOWN G, HEY Y, BOYLE JM AND SANTIBANEZ-KOREF M. (1993). Thirteen dinucleotide repeat polymorphisms on chromosome 6. *Hum. Mol. Genet.*, **2**, 2196.

ORPHANOS V, SANTIBANEZ-KOREF M, MCGOWN G, HEY Y, RACKSTRAW C AND BOYLE JM. (1994*a*). Physical mapping of 43 STSs to human chromosome 6. *Genomics*, **20**, 301-304.

ORPHANOS V, MCGOWN G, HEY Y, BOYLE JM AND SANTIBANEZ-KOREF M. (1995). Proximal 6q, a region showing allele loss in primary breast cancer. *Br. J. Cancer*, **71**, in press.

OSBORNE RJ AND LEECH V. (1994). Polymerase chain reaction allelotyping of human ovarian cancer. *Br. J. Cancer*, **69**, 429-438.

PEJOVIC T, HELM S, MANDAHL N, BALDETORP B, ELMFORS B, FLODERUS U-M, FURGYIK S, HELM G, HIMMELMANN A, WILLEN H AND MITELMAN F. (1992). Chromosome aberrations in 35 primary ovarian carcinomas. *Genes Chrom. Cancer*, **4**, 58-68.

SAITO S, SAITO H, KOOI S, SAGAE S, KUDO R, SAITO J, NODA K AND NAKAMURA Y. (1992*a*). Fine-scale deletion mapping of the distal long arm of chromosome 6 in 70 human ovarian cancers. *Cancer Res.*, **52**, 5815-5817.

SAITO S, OKUI K, TOKINO T, OSHIMURA M AND NAKAMURA Y. (1992*b*). Isolation and mapping of 68 RFLP markers on human chromosome 6. *Am. J. Hum. Genet.*, **50**, 65-70.

SAMBROOK J, FRITSCH EF AND MANIATIS T. (1989). *Molecular Cloning: A Laboratory Manual*, 2nd edn. Cold Spring Harbor Laboratory Press: Cold Spring Harbor, NY.

SANTIBANEZ-KOREF M, ORPHANOS V AND BOYLE JM. (1993). Rapid determination of sequences flanking microsatellites using dephosphorylated cloning vectors. *Trends Genet.*, **9**, 43.

THOMPSON FH, EMERSON J, ALBERTS D, LIU Y, GUAN X-Y, BURGESS A, FOX S, TAETLE R, WEINSTEIN R, MAKAR R, POWELL D AND TRENT J. (1994). Cloncal chromsome abnormalities in 54 cases of ovarian carcinoma. *Cancer Genet. Cytogenet.* **73**, 33-45.

VOLZ A, BOYLE JM, CANN HM, COTTINGHAM RW, ORR HT AND ZIEGLER A. (1994). Report of the 2nd International Workshop on Human Chromosome 6. *Genomics*, **21**, 464-472.

WEISSENBACH J, GYAPAY G, DIB C, VIGNAL A, MORISSETTE J, MILLASSEAU P, VAYSSEIX G AND LATHROP M. (1992). A second-generation linkage map of the human genome. *Nature*, **359**, 794-801.

ZHENG J, ROBINSON WR, EHLEN T, YU MC AND DUBEAU L. (1991). Distinction of low grade from high grade human ovarian carcinomas on the basis of losses of heterozygosity on chromosomes 3, 6, and 11 and HER-2/*neu* gene amplification. *Cancer Res.*, **51**, 4045-4051.